# Adjustment of Assessors’ First Impressions Differs by Student Ethnicity

**DOI:** 10.5334/pme.2196

**Published:** 2025-12-09

**Authors:** Joris J. Steinmann, Inge Otto, Marise Ph. Born, Eva Derous, Andrea M. Woltman, Walter W. van den Broek, Karen M. Stegers-Jager

**Affiliations:** 1 Institute of Medical Education Research Rotterdam, Erasmus MC University Medical Centre Rotterdam, Rotterdam, The Netherlands; 2 Department of Psychology, Education and Child Studies, Erasmus University Rotterdam, Rotterdam, The Netherlands; 3 Optentia and Faculty of Economic and Management Sciences, North-West University, Mahikeng, South Africa; 4 Department of Work Organization and Society, Ghent University, Ghent, Belgium; 5 Radboudumc Health Academy, Radboud University Medical Centre, Nijmegen, The Netherlands

## Abstract

**Introduction:**

Ethnic minority medical students often receive lower grades than their majority counterparts, especially in workplace-based assessments. To unravel this phenomenon of “differential attainment”, we explore assessor bias focusing on how first impressions of students from ethnic minority groups may be adjusted differently compared to majority students when their performance changes.

**Methods:**

In an online experiment with an incomplete block design we created near-identical videos showcasing varying performances of medical students during history-taking. Eighty-one physicians were randomly assigned to watch four different videos each, two with ascending performances (poor start, good ending) and two with descending performances (good start, poor ending), featuring one ethnic minority actress (Turkish or Moroccan origin) and one ethnic majority actress (Dutch origin) as students. We evaluated ethnicity-related differences in first impression ratings (after 60 seconds), final ratings (after 5 minutes), and rating changes.

**Results:**

No significant differences in first impression ratings were found between ethnic groups. Ethnic minority students received higher final ratings than ethnic majority students for ascending performances, but only when rated by residents and not specialists. Finally, rating changes between first impressions and final ratings were larger for ethnic minority than ethnic majority students, for both ascending and descending performances.

**Discussion:**

Our results do not show evidence of assessor bias in first impressions being an explanation for ethnicity-related differential attainment. However, our finding that rating changes were larger for minority than majority students could indicate that they are generally more scrutinized by assessors, which may affect them positively, but also negatively.

## Introduction

Imagine an ethnic minority medical student starts off poorly in a workplace-based assessment (WBA) during their traineeship, but recovers him or herself and ends up performing well, or vice versa. Will assessors adjust their first impressions formed within the first minute in the same way as for ethnic majority students? In this study we explore the role of first impressions and assessor bias in changing student performances in an attempt to unravel the not yet understood phenomenon of ‘differential attainment’. This term refers to the finding that medical students from ethnic minority groups perform less well when compared to majority peers [1,2,3,4,5,6,7], which appears to be particularly prevalent in WBAs. WBAs are widely used to assess students’ readiness for clinical practice by observing and judging their performances in a realistic learning and working environment during traineeships [8]. However, WBAs’ subjective nature [9] and susceptibility to cognitive bias may disproportionately disadvantage the growing number of minority students [10]. This issue has serious implications –not only for individual students, but also for society– since a representative medical workforce is essential for equitable, high-quality healthcare [11].

Since research has consistently shown that differential attainment in WBAs can not be fully explained by prior (pre-clinical) attainment [4,5,7,10], the possibility of assessor bias warrants further investigation. So far, research findings on assessor bias in medical education have been mixed, with multiple Dutch studies highlighting the possibility of implicit discriminatory processes [12,13,14], while several retrospective studies looking at exam data have found little to no evidence of ethnic bias [3,7,15]. The two studies on ethnicity-related assessor bias using controlled experimental conditions revealed either no bias [16] or inconsistent results across different student performance levels [17]. It is noteworthy that these studies focused on stable student performances at borderline [16] or fail/borderline/passing [17] level, and did not consider first impressions.

Dual process theory offers a general theoretical framework about people’s reasoning and decision-making and can help explain how students’ ethnic minority status could affect assessors’ information processing when forming a first impression [18,19,20]. This theory distinguishes two information processing modes: a fast and automatic mode (System 1) and a deliberate, conscious mode (System 2). Relying on System 1 processes, first impressions are made quickly, unconsciously and require few cognitive resources [18,21,22]. Although first impressions can exhibit surprising accuracy [23,24,25], they typically use mental shortcuts (heuristics), which are prone to cognitive biases. Such biases include stereotype activation and prejudiced attitudes [26,27] and might thus result in lower first impression ratings for ethnic minority students [18]. A heuristic that helps to explain how first impressions impact on final judgments is the so-called anchoring effect [28] which proposes that in a certain decision-making procedure (such as assessments), salient information is cued first (like appearance), and subsequently may serve as an anchor for the final judgment. Bias may result from failure to deviate from the anchor in making final judgments [29], which depends on the assessors’ ‘feeling of rightness’, or confidence in their initial response [30]. We suggest that a student’s ethnic minority background may serve as a powerful cue in forming first impressions, potentially leading to greater confidence in those impressions and less adjustment over time. This effect may be especially visible when student performance changes—either ascending (poor start, good ending) or descending (good start, poor ending).

Early studies investigating first impressions in medical education WBAs –not taking student ethnicity into account– revealed that assessors’ first impressions could play a role in explaining variability in final judgments [31], that assessors were willing to change their judgments in case of varying performances [32] and that differences between first impressions and final ratings were larger for descending than for ascending student performances [32]. This study is the first to investigate whether assessors form different first impressions of ethnic minority and majority medical students in WBAs, whether and how these first impressions are related to final ratings, and whether findings are different for ascending and descending student performances. In an exploratory manner we also examine assessor factors that might affect reliance on System 1 processes, including social dominance orientation, [18] (perceived) level of experience [18], and Need For Cognitive Closure (NFCC) [33], a personality characteristic related to being decisive and closed-minded, as increasing factors, and one’s motivation to respond without prejudice as a decreasing factor [18].

We posed the following research questions:

1. Are assessors’ first impressions and accompanying confidence, their final ratings, and/or differences between first impression and final ratings influenced by students’ ethnicity, and does this differ for ascending and descending student performances?
2. Are there associations between ratings of minority and majority students and assessors’ personal characteristics?

## Methods

### Study Design

We used a randomized, double blinded, online, incomplete block design to evaluate ethnicity-related differences in first impression ratings (after 60 seconds), final ratings (after 5 minutes), rating changes (final ratings – first impressions) and raters’ confidence in first impression ratings for both ascending and descending student performances.

### Participants

Participants were physicians (specialists and residents) from Erasmus Medical Center (MC) in Rotterdam and its affiliated hospitals. The inclusion criterion was that they assess medical students as part of their function, therefore, we invited a random sample of our registered assessors, representing different specialties, to participate. Their recruitment was conducted via email and participation in the study was entirely voluntary, devoid of any financial incentives. To mitigate potential biases in responses, we devised a cover story that framed the investigation as an inquiry into potential differences between specialists and residents when evaluating students. This approach aimed to maintain participant impartiality and encourage candid responses and was approved by the Medical Ethics Review Committee of Erasmus MC. Following the completion of the experiment, participants were informed about the actual research goal and were given the opportunity to reaffirm or withdraw their consent to be included in the study.

### Measures and procedures

#### Scripted videos

We created videos of scripted medical student performances with a physician-script writer and the videos were checked by a professional script writer for natural language use. Using actors for all roles, the videos depicted a medical student and a patient in the history-taking part of a consultation. To examine the role of first impressions in changing performance levels, we wrote four different scripts, two with ascending performances (poor start, good ending) by the student and two with descending performances (good start, poor ending). The patient complaints were a cough (script A), stomach complaints (script B), a headache (script C), and tiredness (script D), four common medical issues, with cases scripted that were comparable in terms of complexity to fit the level of first-year Master students. History-taking was deliberately chosen for the scripts since less well-defined competences, such as communication may lend themselves more to assessor bias than technical skills [6].

The scripts, which also included non-verbal cues, were reviewed by a panel of six experienced physicians to make sure that they were realistic and showed the intended performance changes (the results of the review session can be found in Appendix A). After minor revisions, all scripts were filmed twice, once with an ethnic majority actress (Dutch origin) and once with an ethnic minority actress (Moroccan or Turkish origin) playing a medical student, resulting in a total of 8 videos. We performed a detailed comparison of the videos after filming to ensure their similarity in both verbal and non-verbal behavior, e.g. silences, tone of voice. We filmed the videos with four different actresses (2 of ethnic majority, 2 of ethnic minority), so participants watched every actress only once. All participants watched every script once (4 videos): two ascending and two descending performances, but with different ethnicities across groups. Participants were randomly placed in two groups by the questionnaire software Limesurvey. Within each group participants were randomly assigned to one of four video orders that were created using Latin Squared Counterbalancing. An overview of the study design is presented in Table 1.

**Table 1:** Study Design.


STUDENT ETHNICITY	ASCENDING PERFORMANCE		DESCENDING PERFORMANCE	
	
	MAJORITY	MINORITY	MAJORITY	MINORITY

Group 1	Script A (video 1)	Script B (video 4)	Script C (video 5)	Script D (video 8)

Group 2	Script B (video 3)	Script A (video 2)	Script D (video 7)	Script C (video 6)


Notes. Scripts: A cough, B stomach complaints, C headache complaints, D tiredness. Group 1 watched videos 1, 4, 5, and 8. Group 2 watched videos 2, 3, 6, and 7. Within both groups, videos were presented in four different orders.

#### Performance Rating and Confidence

Participants watched the four videos they were assigned online. Each video stopped after 60 seconds (about 1/5^th^ of total length) and participants were asked to give their first impression rating of the performance so far (on a scale from 1 to 10, with 1 = poor and 10 = excellent, commonly used in the Dutch education system), as well as their confidence about said score (“I am … about my rating just given”, rated on a scale from 1 “very uncertain” to 7 “very confident”). Afterwards, they watched the remainder of the video (ca. 4 minutes) and were then asked to give their final rating (again between 1 and 10). The rating scale corresponds to the grades actual medical students receive during their studentship as part of their medical education. We decided to use a 60 second timeframe for the first impression because this is in line with a previous study on first impressions and assessment in medical education [31], and because the video-experiment of Carney et al. [34] showed that 60 seconds (rather than 5, 20, 45 or 300s) gave the optimal ratio between accuracy and exposure time to form a first impression.

#### Student Ethnicity

As the concept of “ethnicity” is complex, politically charged, and also context specific [35], we consider it important to explicitly state how we defined and operationalized the concept. In this study, we followed the recently introduced classification by origin of Statistics Netherlands, according to which an individual belongs to an ethnic minority group if the individual was either born abroad him/herself (“migrant”) or of whom at least one parent was born abroad (“child of migrant(s)”). An important element in the new classification refers to so-called ‘traditional countries of origin’- countries with historically strong migration ties with the Netherlands – which include Turkey, Morocco, Surinam, the Dutch Caribbean and Indonesia. In our study, the majority students were represented by actresses of Dutch origin. The ethnic minority students were represented by actresses of Turkish or Moroccan origin, which was visible in their ethnic name and appearance (e.g., headscarf).

We opted for students of Moroccan/Turkish origin because, amongst other reasons, the Turkish and the Moroccan minority populations are two of the four largest ethnic minority groups in The Netherlands [36], suffering from ethnic discrimination in education [5] as well as in the labour market, in particular when wearing a headscarf [37]. We deliberately chose females as students as they are the largest gender group in medical school, and to exclude the unique effects of students’ gender in evaluations.

#### Additional Variables and Debriefing

Following the videos, the participants answered demographic questions about their age, gender, country of origin function, medical specialty, experience with assessing the history-taking skills of medical students, and number of years of experience in clinical practice.

##### Need for cognitive closure (NFC)

To measure NFC, we used Roets and Van Hiel’s [38] validated 15-item Need for Cognitive Closure questionnaire. An example item was “I don’t like situations that are uncertain.” Answers were provided on a 6-point Likert-type scale, ranging from 1 “completely disagree” to 6 “completely agree.”

##### Social dominance orientation (SDO)

SDO was measured using the 6-item Dutch version of the Social Dominance Scale by Onraet et al. [39]. An example item was “This country would be better off if we cared less about how equal all people are.” The statements were rated on a 5-point Likert-type scale from 1 “strongly disagree” to 5 “strongly agree.”

##### Internal and external motivation to respond without prejudice (IMS/EMS)

Our study used an adapted version of the Internal Motivation to Respond Without Prejudice Scale (IMS) and the External Motivation to Respond Without Prejudice Scale (EMS). Originally developed by Plant and Devine [40], Derous, Ryan and Serlie [41] translated and validated a Dutch version of these scales. The scales each consist of 5 items and participants use 5 point-Likert scales ranging from 1 “strongly disagree” to 5 “strongly agree.” An example item of the original scale was “I attempt to act in nonprejudiced ways toward Black people because it is personally important to me.” In the Dutch version, “Black people” was replaced by “people with a Moroccan/Turkish migration background”.

Finally, the participants were debriefed, fully informed about the goals of the study, and were then asked to give or withdraw their consent to be included in the study.

### Analysis

Linear Mixed Model (LMM) analyses in SPSS version 28 were performed to account for potentially unequal group sizes, as well as to correct for differences between scripts watched by the two groups. Beforehand, a power analysis was performed with G*Power to determine the sample size needed. As this tool could not be used for LMMs, the power analysis was performed under the assumption of the similar repeated measures ANOVA [42]. Assuming small to medium effect sizes (f = 0.17), based on results of the meta-analysis by Woolf et al. [35], a total sample size of 70 participants was needed for a power of .80 and an alpha of .05 [42].

#### First Impression Ratings and Final Ratings

The First Impression Ratings of ascending performances (poor start) and descending performances (good start) were analyzed separately. To analyze differences in the outcome variable First Impression Ratings, student ethnicity was added as a fixed within-subjects factor. Next, we investigated the differences in Final Ratings of ascending performances (good ending) and descending performances (poor ending). Again, student ethnicity was added as a fixed within-subjects factor.

#### Rating Changes

To analyze a potential anchoring effect, we first computed the mean absolute difference between first impression and final rating as the outcome variables for both ascending and descending performances separately. Then, student ethnicity was again added to the model as the fixed within-subjects factor.

#### Confidence in First Impression Ratings

To support the findings of the proposed anchoring effect, we examined whether the assessors’ confidence in their first impression ratings was higher when rating ethnic minority students than ethnic majority students, in both ascending and descending performances separately. To analyze differences in the outcome variable confidence, student ethnicity was once again added as a fixed within-subjects factor.

#### Differences Between Ascending and Descending Performances

If ethnicity-related differences in final ratings or in rating changes were found, we would expand the previous analyses and add performance level (ascending/descending) as a second within-subjects variable.

#### Assessor Factors

As an exploratory analysis, we used Pearson’s correlation coefficient to measure all bi-variate correlations of the assessor factors function, experience with assessing medical students, NFC, SDO, IMS, and EMS with the outcome variables.

## Results

### Descriptive Statistics

Having received the invitations, 136 participants gave consent and started the questionnaire. Of these, 95 reached the first question and 81 finished and gave their repeated consent after being debriefed. When asked about their perceived purpose of the study (right after watching the videos), one out of the 81 participants (1.2%) appeared to have guessed correctly. Excluding this participant did not change any of the results. No other participants had to be excluded from the dataset either, as all came from the target population (specialists and residents) and seemed to have followed the instructions (e.g., no unusual response times). The two groups did not differ significantly on number of dropouts (27 (group 1) vs 28 (group 2), p = 0.60) nor on demographics (see Appendix B, Table B1). Seven participants (8.6%) reported a country of origin outside Europe. Excluding them did not change the results.

### Outcome measures

#### First Impressions and Final Ratings

**Figure 1:**
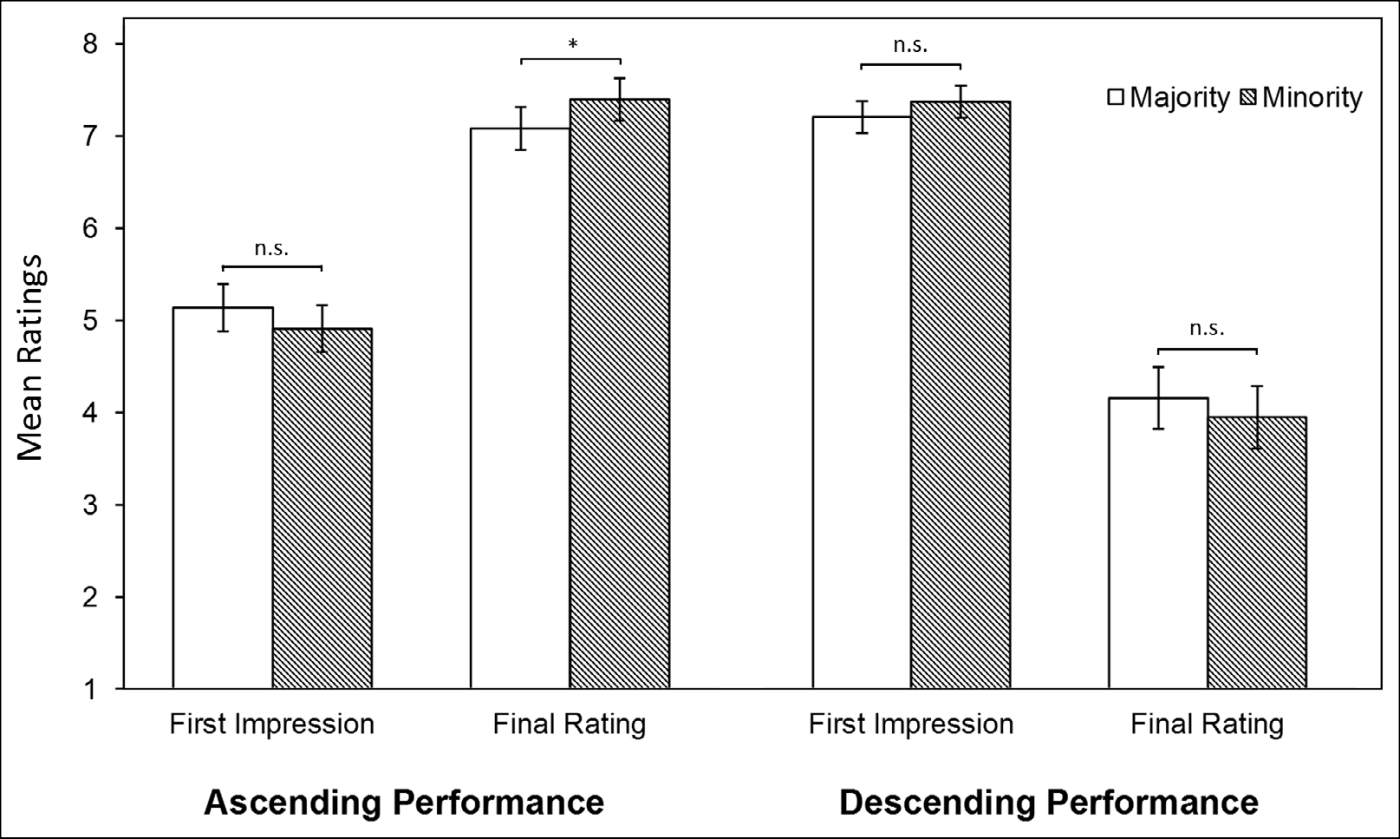
LMM Estimates of First Impression and Final Ratings. Notes. **p < .01, *p < .05, n.s. not significant. LMM estimates are controlled for script differences.

Looking at the first impression ratings of the ascending performance (poor start), mean scores for ethnic minority students were 4.91 (SD = 0.13) and for ethnic majority students 5.14 (SD = 0.13); this difference was not statistically significant, *F*(1,83) = 2.68, *p* = .11 (see Figure 1). For the descending performance (good start), mean first impression ratings were 7.37 (SD = 0.09) for ethnic minority students and 7.21 (SD = 0.09) for ethnic majority students; this difference was not significant either, *F*(1,79) = 2.11, *p* = .15. All estimates are controlled for potential differences in scripts (see Appendix C, Table C1).

For the final rating of the ascending performances (good ending), ethnic majority students were rated significantly lower (M = 7.09, SD = .12) than ethnic minority students (M = 7.40, SD = .12), *F*(1,79) = 5.99, *p* = .02 (see Figure 1, Appendix C). Post hoc analyses showed a significant interaction effect of student ethnicity with the assessors’ function, *F*(1,78) = 6.81, *p* = .01, revealing that this higher rating of ethnic minorities was only the case when the students were rated by residents (M_maj_ = 6.95, SD_maj_ = .16; M_min_ = 7.56, SD_min_ = .16, *d* = 0.56), not specialists (M_maj_ = 7.25, SD_maj_ = .17; M_min_ = 7.21, SD_min_ = .17). For the descending performances (poor ending), mean scores for the ethnic minority students were 3.95 (SD = .17) and for the ethnic majority students 4.16 (SD = 0.17); this difference was not significant, *F*(1,79) = 2.362, *p* = .13. Therefore, the additional analysis including performance level was considered redundant. Again, all estimates were controlled for potential differences in scripts (see Appendix C, Table C1).

#### Rating Changes

**Figure 2:**
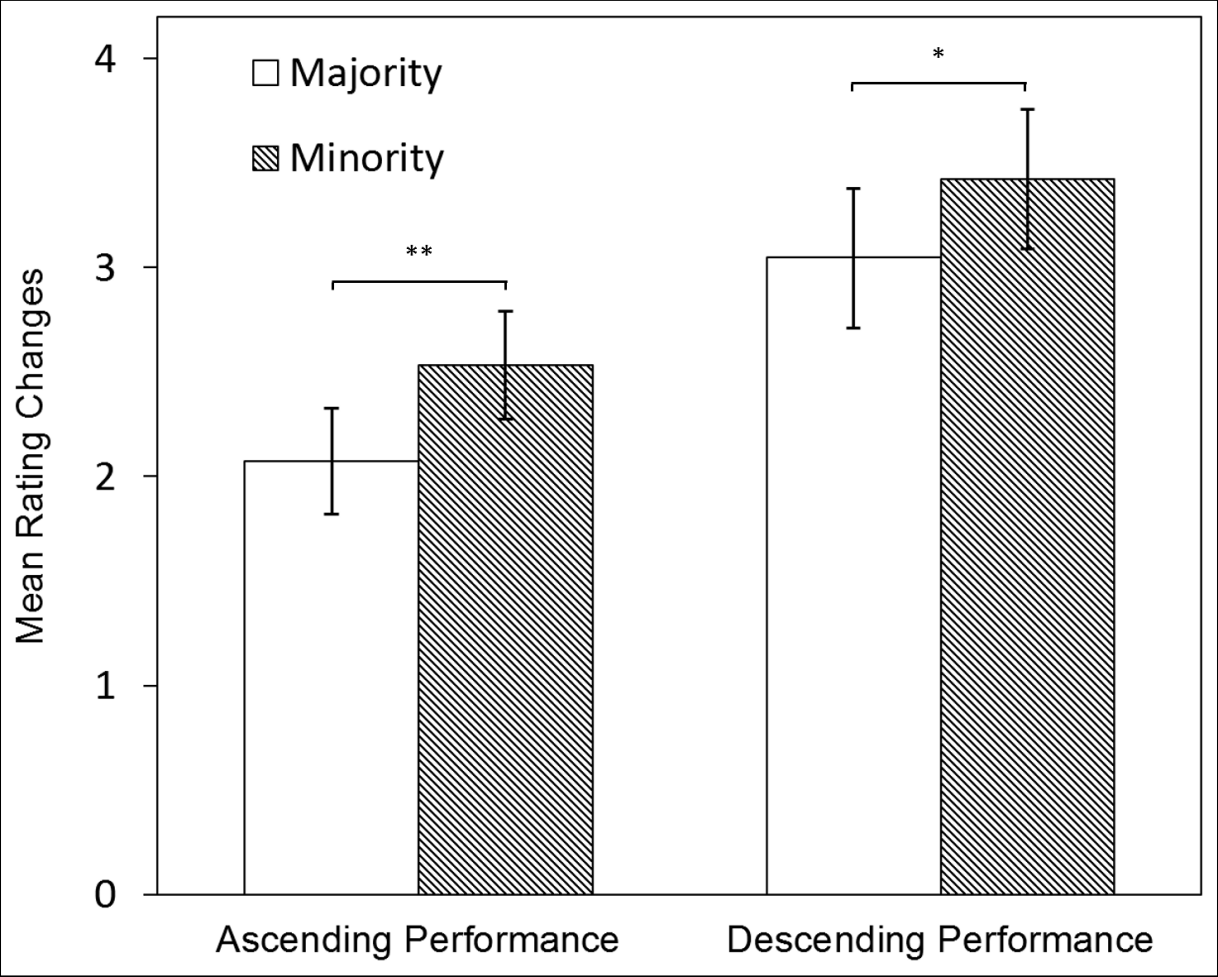
Rating Changes: LMM Estimates of Changes Between First Impressions and Final Ratings. Notes. **p < .01, *p < .05. LLM estimates are controlled for script differences.

Examining the ascending performances, we found that the rating changes were significantly smaller for ethnic majority students (M = 2.07, SD = .13) than for ethnic minority students (M = 2.53, SD = .13), *F*(1,83) = 11.65, *p* < .001, *d* = .37. The descending performances showed a similar result, with significantly smaller rating changes for ethnic majority students (M = 3.05, SD = .17) than for ethnic minority students (M = 3.442, SD = .17), *F*(1,79) = 5.541, *p* = .02, *d* = .24. As both effects were statistically significant, we expanded our analysis by adding performance level. Additionally to ethnicity, performance level (ascending/descending) was found to be significant, *t*(247) = –5.65, *p* < .001, indicating that rating changes were larger for descending performances than ascending performances (see Figure 2). The interaction effect between ethnicity and performance level was not significant, *t*(247) = –.51, *p* = .61. Once more, all estimates were controlled for potential differences in scripts (see Appendix C, Table C1).

#### Confidence in First Impression Ratings

No significant differences in assessors’ confidence in their first impression ratings for ethnic minority and majority students were found, neither within ascending performances M_maj_ = 5.10, SD_maj_ = .12; M_min_ = 5.28, SD_min_ = .12), *F*(1,79) = 2.61, *p* = .11, nor descending performances M_maj_ = 5.32, SD_maj_ = .10; M_min_ = 5.39, SD_min_ = .10), *F*(1,79) = 0.63, *p* = .43.

#### Assessor Factors (RQ3)

Bi-variate correlations of all items at the individual level can be found in Appendix D.

Assessors’ concerns about prejudice (IMS and EMS) and social dominance orientation (SDO) did not show any significant correlations with the outcome variables. The need for cognitive closure(NFC) showed significant positive correlation with final ratings of ethnic minority students for both ascending and descending performances. For majority students, NFC correlated significantly and positively with the final ratings and negatively with the rating changes in descending performances.

## Discussion

The goal of this study was to explore whether and how first impressions are related to final ratings for both ethnic minority and majority students in WBAs. There were several notable findings. First, no significant differences in first impression ratings were found between ethnicities, neither for poor, nor for good starts. Second, ethnic minority students received significantly higher final ratings than ethnic majority students when looking at the ascending performances, but only when rated by residents. For the final ratings of descending performances, no significant differences were found. Third, rating changes between first impressions and final ratings were larger for descending than ascending performance and, within both performance levels, the rating changes were larger for ethnic minority than ethnic majority students. The interaction between performance level and ethnicity was not significant, indicating that the effect of ethnicity was equal for both performance levels. Fourth, we found no student ethnicity-related differences in assessors’ confidence in first impression ratings. The assessor personality factor NFC correlated negatively with the rating changes of ethnic majority students in descending performances, suggesting that assessors with a high(er) need for closure were less likely to negatively adjust their scores for majority students.

Assessors’ first impressions were not influenced by students’ ethnicity. Within the theoretical framework of dual process theory, explanations could be twofold. First, assessors may not hold any biases towards ethnic minorities. This, however, would be as admirable as it is remarkable since multiple studies within the Dutch medical education context report ethnic minority students and residents experiencing differential treatment in their educational program [12,13,43]. Note, however, that although not significant, we noticed some tendency to score ethnic minority students lower than their majority counterpart in poor performances (poor start and poor ending). A second, probably more realistic explanation, would be that assessors may still hold some biases, but instead of relying solely on their automatic System 1, they were able to self-regulate behavioral impulses and ‘override’ them through their System 2 when giving their first impression ratings [44]. This may have been enabled through the study design, where assessors were given 60 seconds of the student performance to form a first impression, as well as time to consider it. With this choice of time to form a first impression, this study followed the reported optimal ratio between accuracy and exposure time [34]. Considering our findings, it appears as though 60 seconds are not only enough to form an accurate impression but may also be enough to overcome biased first impressions. Furthermore, just the act of making an explicit rating of their first impression may have activated the more deliberate, conscious System 2. It is worth noting that Wood recently showed that assessors adjusted their judgments based on students’ performances both after implicitly-formed, i.e. in a realistic setting, and explicitly-formed first impressions [45]. Assessors’ abilities to self-regulate would also be in line with Yeates et al. [16], who found that assessors did activate stereotypes, but apparently were able not to let them influence their ratings.

When looking at final ratings, minority students did not receive significantly lower scores. This suggests that assessor bias may not be responsible for the real-world differential attainment and supports previous field studies [3,15] and comparable experiments [16] that came to the same conclusion. Noticeably, minority students even received higher final ratings for the ascending performance, however only when rated by the younger and less experienced residents. This generational gap could reflect a social desirability bias by resident assessors, referring to the tendency of participants to choose responses they believe to be more socially desirable (i.e. rating minority students higher) [46]. This is supported by a significant, negative correlation between EMS and age, which suggests that the younger assessors care more about how prejudiced they are perceived by others.

Rating changes between first and final ratings were larger for ethnic minority than majority students. Furthermore, our participants did not particularly maintain or reveal more confidence in their first impression ratings of ethnic minority students, contradicting the suggested anchoring effect. It still seems plausible that the assessors did take the ethnicity of the minority student as a strong cue. However, instead of serving as an unconscious anchor, this stronger cue may have made the assessors more alert to the performance of the student. This may reflect a form of tokenism, a phenomenon first described by Kanter [47]. She highlighted how underrepresented groups (in her case women and in the present case ethnic minorities) can often be treated as tokens – symbolic representatives – rather than as fully integrated and valued members of the organization. According to tokenism, ethnic minority students would attract disproportional attention [47], which in our study may have led to exaggerated differences between them and the ethnic majority students [48]. The finding that rating changes were larger for descending than ascending performances was in line with Wood et al. [32], who suggested that this negativity bias may be out of fear for wrongfully passing a student who should not become a doctor. The absence of a significant interaction effect suggests there is no “double-disadvantage effect” for minority students, c.f. Nieminen et al. [49].

An alternative explanation may be found in attribution theory [50]. Assessors may have attributed the success of ethnic minority students in ascending performance scenarios to internal factors (e.g., exceptional ability, effort) despite potential obstacles and negative stereotypes they are expected to face. This attributional augmenting involves amplifying the perceived significance of internal factors, which may lead to higher ratings for ethnic minority students compared to ethnic majority students. The positive performance in the face of potential challenges may be seen as particularly noteworthy, contributing to this augmenting effect. The descending performance, on the other hand, would strengthen pre-existing, negative stereotypes and may contribute to a penalty in the rating of ethnic minority students (i.e., attributional discounting). Although in a different context, similar results have been described by Derous et al. [51]. They found that highly qualified ethnic minority (Arabic) applicants received more favorable ratings for highly cognitive-demanding jobs than ethnic majority applicants (attributional augmenting) but, at the same time, lower qualified Arabic profiles were not preferred for low cognitive-demanding jobs (attributional discounting).

### Strengths and Limitations

This study is the first to explore the formation of first impressions of ethnic minority and majority medical students and their influence on the final ratings they receive when performances change. It holds strong internal validity with its double blinded, randomized, within-subjects design and a successful manipulation of different performance levels. Each assessor watched four scripted videos – carefully reviewed for content and (non)verbal similarity – of different, deliberately chosen, medical issues increasing ecological validity and preventing participant fatigue. Furthermore, all participants were doctors who evaluate students as part of their job. It is the same population that is part of the observed, actual differential attainment, underlining this study’s strong external validity as well.

Our study also has some limitations. The first drawback of our experimental design is its simulated form as opposed to real world observations. Second, some of the observed variations in ratings may be due to real variation in performance or other (visible) differences between individuals rather than perceptions of ethnicity alone, and small differences may have gone undetected. Nevertheless, it is encouraging that those results that could be compared to previous studies are in line with their findings. Third, our study only featured two female actors of Turkish/Moroccan origin to represent minority students. Although this choice was deliberate, given their reported underperformance during medical school [5] and underrepresentation in the medical workforce [52], the broader group of ethnic minority students is more diverse, and different biases may apply. Furthermore, our findings may not generalize to all students of Turkish/Moroccan origin, nor to male students. Grouping people of different cultures and nations as ‘minorities’ is a limitation as differences within cultures may exceed those between cultures. Nonetheless, we deliberately chose a specific ethnic minority group which is highly relevant in our context to test our suggested mechanism. To assess generalizability and repeatability, we encourage replication in similar and different contexts using male and female students from ethnic groups relevant to those contexts. If sample sizes would allow it, future work could expand on our exploratory findings regarding assessors’ characteristics, possibly also considering assessors’ ethnicity and gender.

### Practical Implications

Our findings suggest that the mere fact of having a slightly different skin tone, name and a head scarf does not directly impact student assessment. However, this could also imply that the topic of ethnic bias is more complex, and ethnic differences may manifest as more subtle than the direct, visual observations – through accents, communication styles, social norms and values. Building on our findings, future studies could explore whether ethnicity in a broader term would show different results. Future studies may also want to explore the role of assessors’ written feedback [53] or prior performance information [54] in explaining real-world differential attainment.

A second, practical implication for students is that a poor first impression can be overcome as assessors appear to recognize and reward upward performance trends. However, a strong start doesn’t guarantee continued success. Notably, rating changes were more pronounced for minority students, suggesting they may face greater scrutiny. While this can benefit ascending performers, it may also increase stress and discomfort, potentially leading to underperformance. Prior research supports this observation, showing minority students are more prevention-focused, perceive more unfair treatment, and have lower trust in supervisors [55]. One solution to unjustified rating differences would be clear rating criteria that are known to both students and assessors, minimizing the ambiguity and subjectivity of WBAs.

Another proposed solution in Dutch medical schools is eliminating grades and distinctions like ‘cum laude’ to reduce performance pressure and stress. A third solution could be adopting programmatic assessment, which offers a holistic view of student progress and therefore has the promise of enabling equity [56].

## Conclusion

In conclusion, our study found that both poor and good first impressions of medical students will be overcome if the subsequent performance changes. Furthermore, we did not find direct evidence that ethnic assessor bias is the reason for real-world differential attainment, as first impression and final ratings were not lower for ethnic minority students. However, assessors appeared to be more sensitive to performance changes of ethnic minority students, with larger differences between first impression and final ratings. Future efforts to minimize the potential of ethnic bias when rating medical students could focus on reducing subjectivity with clear rating criteria, reducing stress factors such as honorary distinctions, or shifting towards programmatic assessments.

## Data Accessibility Statement

The data generated during the current study are not publicly available due to the sensitivity of the data but are available from the corresponding author on reasonable request.

## Additional File

The additional file for this article can be found as follows:

10.5334/pme.2196.s1 Appendices. Appendix A to D.

## References

[1] Haq I, Higham J, Morris R, Dacre J. Effect of ethnicity and gender on performance in undergraduate medical examinations. Med Educ. 2005;39(11):1126–8. DOI: 10.1111/j.1365-2929.2005.02319.x 16262808

[2] Lidell MJ, Koritsas S. Effect of medical students’ ethnicity on their attitudes towards consultation skills and final year examination performance. Med Educ. 2004;38(2):187–98. DOI: 10.1111/j.1365-2923.2004.01753.x 14871389

[3] McManus IC, Richards P, Winder BC, Sproston KA. Final examination performance of medical students from ethnic minorities. Med Educ. 1996;30(3):195–200. DOI: 10.1111/j.1365-2923.1996.tb00742.x 8949553

[4] Stegers-Jager KM, Brommet FN, Themmen APN. Ethnic and social disparities in different types of examinations in undergraduate pre-clinical training. Adv Health Sci Educ Theory Pract. 2016;21(5):1023–46. DOI: 10.1007/s10459-016-9676-7 27015959 PMC5119835

[5] Stegers-Jager KM, Steyerberg EW, Cohen-Schotanus J, Themmen AP. Ethnic disparities in undergraduate pre-clinical and clinical performance. Med Educ. 2012;46(6):575–85. DOI: 10.1111/j.1365-2923.2012.04265.x 22626049

[6] Wass V, Roberts C, Hoogenboom R, Jones R, Van der Vleuten C. Effect of ethnicity on performance in a final objective structured clinical examination: qualitative and quantitative study. BMJ. 2003;326(7393):800–3. DOI: 10.1136/bmj.326.7393.800 12689978 PMC153100

[7] Woolf K, Haq I, McManus IC, Higham J, Dacre J. Exploring the underperformance of male and minority ethnic medical students in first year clinical examinations. Adv Health Sci Educ Theory Pract. 2008;13(5):607–16. DOI: 10.1007/s10459-007-9067-1 17487565

[8] ten Cate TJO, Snell L, Carraccio C. Medical competence: the interplay between individual ability and the health care environment. Med Teach. 2010;32(8):669–75. DOI: 10.3109/0142159x.2010.500897 20662579

[9] Gingerich A, Kogan J, Yeates P, Govaerts M, Holmboe E. Seeing the ‘black box’ differently: assessor cognition from three research perspectives. Med Educ. 2014;48(11):1055–68. DOI: 10.1111/medu.12546 25307633

[10] Esmail A, Roberts C. Academic performance of ethnic minority candidates and discrimination in the MRCGP examinations between 2010 and 2012: analysis of data. BMJ. 2013;347:f5662. DOI: 10.1136/bmj.f5662 24072882 PMC3898419

[11] Stanford FC. The importance of diversity and inclusion in the healthcare workforce. J Natl Med Assoc. 2020;112(3):247–49. DOI: 10.1016/j.jnma.2020.03.014 32336480 PMC7387183

[12] Tjitra JJ, Leyerzapf H, Abma TA. “Dan blijf ik gewoon stil”: ervaringen van allochtone studenten met interculturalisatie tijdens de opleiding Geneeskunde. TS Medisch Onderwijs. 2011;30(6):292–301. DOI: 10.1007/s12507-011-0067-z

[13] Leyerzapf H, Abma TA, Steenwijk RR, Croiset G, Verdonk P. Standing out and moving up: performance appraisal of cultural minority physicians. Adv Health Sci Educ Theory Pract. 2015;20(4):995–1010. DOI: 10.1007/s10459-014-9577-6 25549932

[14] Isik U, Wouters A, Verdonk P, Croiset G, Kusurkar RA. “As an ethnic minority, you just have to work twice as hard.” Experiences and motivation of ethnic minority students in medical education. Perspect Med Educ. 2021;10(5):272–8. DOI: 10.1007/s40037-021-00679-4 34515955 PMC8505584

[15] Denney ML, Freeman A, Wakeford R. MRCGP CSA: are the examiners biased, favouring their own by sex, ethnicity, and degree source? Br J Gen Pract. 2013;63(616):e718–25. DOI: 10.3399/bjgp13X674396 24267854 PMC3809424

[16] Yeates P, Woolf K, Benbow E, Davies B, Boohan M, Eva K. A randomised trial of the influence of racial stereotype bias on examiners’ scores, feedback and recollections in undergraduate clinical exams. BMC Med. 2017;15(1):179. DOI: 10.1186/s12916-017-0943-0 29065875 PMC5655938

[17] Brown C, Khavandi S, Sebastian A, Badger K, Westacott R, Reed MWR, et al. The influence of candidates’ race on examiners’ ratings in standardised assessments of clinical practice. Med Teach. 2025;47(3):492–97. DOI: 10.1080/0142159x.2024.2345266 38771961

[18] Derous E, Buijsrogge A, Roulin N, Duyck W. Why your stigma isn’t hired: A dual-process framework of interview bias. Hum Resour Manag Rev. 2016;26(2):90–111. DOI: 10.1016/j.hrmr.2015.09.006

[19] Pryor JB, Reeder GD, Yeadon C, Hesson-McInnis M. A dual-process model of reactions to perceived stigma. J Pers Soc Psychol. 2004;87(4):436–52. DOI: 10.1037/0022-3514.87.4.436 15491270

[20] Ingold PV, Dönni M, Lievens F. A dual-process theory perspective to better understand judgments in assessment centers: The role of initial impressions for dimension ratings and validity. J Appl Psychol. 2018;103(12):1367–78. DOI: 10.1037/apl0000333 30058812

[21] Ambady N. The perils of pondering: Intuition and thin slice judgments. Psychol Inq. 2010;21(4):271–78. DOI: 10.1080/1047840X.2010.524882

[22] Wood TJ. Exploring the role of first impressions in rater-based assessments. Adv Health Sci Educ Theory Pract. 2014;19:409–27. DOI: 10.1007/s10459-013-9453-9 23529821

[23] Kahneman D, Klein G. Conditions for intuitive expertise: a failure to disagree. Am Psychol. 2009;64(6):515–26. DOI: 10.1037/a0016755 19739881

[24] Norman G. Dual processing and diagnostic errors. Adv Health Sci Educ Theory Pract. 2009;14:37–49. DOI: 10.1007/s10459-009-9179-x 19669921

[25] Ambady N, Rosenthal R. Thin slices of expressive behavior as predictors of interpersonal consequences: A meta-analysis. Psychol Bull. 1992;111(2):256–74. DOI: 10.1037/0033-2909.111.2.256

[26] Bodenhausen GV, Wyer RS. Effects of stereotypes in decision making and information-processing strategies. J Pers Soc Psychol. 1985;48(2):267–82. DOI: 10.1037/0022-3514.48.2.267 3981396

[27] Kunda Z, Spencer SJ. When do stereotypes come to mind and when do they color judgment? A goal-based theoretical framework for stereotype activation and application. Psychol Bull. 2003;129(4):522–44. DOI: 10.1037/0033-2909.129.4.522 12848219

[28] Kahneman D. Thinking, fast and slow. New York: Farrar, Straus and Giroux; 2011.

[29] Eroglu C, Croxton KL. Biases in judgmental adjustments of statistical forecasts: The role of individual differences. Int J Forecast. 2010;26(1):116–33. DOI: 10.1016/j.ijforecast.2009.02.005

[30] Thompson VA. Dual process theories: A metacognitive perspective. In: Evans JSBT, Frankish K, editors. In two minds: Dual processes and beyond. New York: Oxford University Press; 2009. pp. 171–95. DOI: 10.1093/acprof:oso/9780199230167.003.0008

[31] Wood TJ, Chan J, Humphrey-Murto S, Pugh D, Touchie C. The influence of first impressions on subsequent ratings within an OSCE station. Adv Health Sci Educ Theory Pract. 2017;22:969–83. DOI: 10.1007/s10459-016-9736-z 27848171

[32] Wood TJ, Pugh D, Touchie C, Chan J, Humphrey-Murto S. Can physician examiners overcome their first impression when examinee performance changes? Adv Health Sci Educ Theory Pract. 2018;23(4):721–32. DOI: 10.1007/s10459-018-9823-4 29556923

[33] Webster DM, Kruglanski AW. Individual differences in need for cognitive closure. J Pers Soc Psychol. 1994;67(6):1049–62. DOI: 10.1037//0022-3514.67.6.1049 7815301

[34] Carney DR, Colvin CR, Hall JA. A thin slice perspective on the accuracy of first impressions. J Res Pers. 2007;41(5):1054–72. DOI: 10.1016/j.jrp.2007.01.004

[35] Woolf K, Potts HW, McManus IC. Ethnicity and academic performance in UK trained doctors and medical students: systematic review and meta-analysis. BMJ. 2011;342:d901. DOI: 10.1136/bmj.d901 21385802 PMC3050989

[36] Statistics Netherlands. Annual Report on Integration 2018. The Hague: Statistics Netherlands; 2018.

[37] Radboud University Nijmegen. Moslima’s kampen met discriminatie op de arbeidsmarkt [Muslim women face discrimination in the labor market]. Radboud University Nijmegen; 2024 11/06/2024.

[38] Roets A, Van Hiel A. Item selection and validation of a brief, 15-item version of the Need for Closure Scale. Pers Individ Dif. 2011;50(1):90–4. DOI: 10.1016/j.paid.2010.09.004

[39] Onraet E, Dhont K, Van Hiel A. The relationships between internal and external threats and right-wing attitudes: A three-wave longitudinal study. Pers Soc Psychol Bull. 2014;40(6):712–25. DOI: 10.1177/0146167214524256 24570118

[40] Plant EA, Devine PG. Internal and external motivation to respond without prejudice. J Pers Soc Psychol. 1998;75(3):811–32. DOI: 10.1037/0022-3514.75.3.811 16055643

[41] Derous E, Ryan AM, Serlie AW. Double jeopardy upon resume screening: When Achmed is less employable than Aisha. Pers Psychol. 2015;68(3):659–96. DOI: 10.1111/peps.12078

[42] Faul F, Erdfelder E, Lang A-G, Buchner A. G* Power 3: A flexible statistical power analysis program for the social, behavioral, and biomedical sciences. Behav Res Methods. 2007;39(2):175–91. DOI: 10.3758/BF03193146 17695343

[43] Isik U. Motivation and academic performance of ethnic minority medical students: ‘Struggling and coping in the path from student to doctor’ [PhD thesis]. Amsterdam: Vrije Universiteit Amsterdam; 2019.

[44] Evans JSBT, Stanovich KE. Dual-process theories of higher cognition: Advancing the debate. Perspect Psychol Sci. 2013;8(3):223–41. DOI: 10.1177/1745691612460685 26172965

[45] Wood TJ, Daniels VJ, Pugh D, Touchie C, Halman S, Humphrey-Murto S. Implicit versus explicit first impressions in performance-based assessment: will raters overcome their first impressions when learner performance changes? Adv Health Sci Educ Theory Pract. 2023;1–14. DOI: 10.1007/s10459-023-10302-2 38010576

[46] Grimm P. Social desirability bias. In: Sheth J, Malhotra N, editors. Wiley International Encyclopedia of Marketing. Part 2. Hoboken, NJ, USA: John Wiley & Sons; 2010. pp. 258–59. DOI: 10.1002/9781444316568.wiem02057

[47] Kanter RM. Men and women of the corporation. New York: Basic Books; 1977.

[48] Niemann YF. Tokenism. In: Naples NA, editor. The Wiley Blackwell Encyclopedia of gender and sexuality studies. Hoboken, NJ, USA: John Wiley & Sons, Ltd; 2016. pp. 1–2. DOI: 10.1002/9781118663219.wbegss678

[49] Nieminen LRG, Rudolph CW, Baltes BB, Casper CM, Wynne KT, Kirby LC. The combined effect of ratee’s bodyweight and past performance information on performance judgments. J Appl Soc Psychol. 2013;43(3):527–43. DOI: 10.1111/j.1559-1816.2013.01033.x

[50] Kelley HH. Attribution in social interaction. In: Jones EE, Kanouse DE, Kelley HH, Nisbett RE, Valins S, Weiner B, editors. Attribution: Perceiving the causes of behavior. Mahwah, NJ, USA: Lawrence Erlbaum Associates, Inc; 1987. pp. 1–26.

[51] Derous E, Nguyen H-H, Ryan AM. Hiring discrimination against Arab minorities: Interactions between prejudice and job characteristics. Hum Perform. 2009;22(4):297–320. DOI: 10.1080/08959280903120261

[52] Mulder L, Wouters A, Akwiwu EU, Koster AS, Ravesloot JH, Peerdeman SM, et al. Diversity in the pathway from medical student to specialist in the Netherlands: a retrospective cohort study. Lancet Reg Health Eur. 2023;35:100749. DOI: 10.1016/j.lanepe.2024.101139 37860636 PMC10583163

[53] Rojek AE, Khanna R, Yim JWL, Gardner R, Lisker S, Hauer KE, et al. Differences in Narrative Language in Evaluations of Medical Students by Gender and Under-represented Minority Status. J Gen Intern Med. 2019;34(5):684–91. DOI: 10.1007/s11606-019-04889-9 30993609 PMC6502922

[54] Humphrey-Murto S, Shaw T, Touchie C, Pugh D, Cowley L, Wood TJ. Are raters influenced by prior information about a learner? A review of assimilation and contrast effects in assessment. Adv Health Sci Educ Theory Pract. 2021;26:1133–56. DOI: 10.1007/s10459-021-10032-3 33566199

[55] van Andel CEE, Born MP, van den Broek WW, Stegers-Jager KM. Student ethnicity predicts social learning experiences, self-regulatory focus and grades. Med Educ. 2022;56(2):211–19. DOI: 10.1111/medu.14666 34543459 PMC9293402

[56] Lucey CR, Hauer KE, Boatright D, Fernandez A. Medical education’s wicked problem: achieving equity in assessment for medical learners. Acad Med. 2020;95(12S):S98–108. DOI: 10.1097/acm.0000000000003717 32889943

