# Supplementary material for: Adjustment of Assessors’ First Impressions Differs by Student Ethnicity
Source: Perspect Med Educ. 2025 Dec 9;14(1):991–1002. doi: 10.5334/pme.2196 (PMC12700149; doi:10.5334/pme.2196)
Supplement: Appendices. — Appendix A to D. [file pme-14-1-2196-s1.pdf]

Appendix A

Validation of student performance changes

With the help of a physician-script writer, we created four different scripts: two with ascending performances (poor start, good ending) by the student and two with descending performances (good start, poor ending). The patient complaints were a cough (script A), stomach complaints (script B), a headache (script C), and tiredness (script D). To validate the intended performance changes and the authenticity of the cases, a panel of six experienced physicians reviewed the scripts. For each script, they were asked to give the student a rating after a relatively short first part ("first impression", which would later be the first 60 seconds of the video) and a final rating for the full performance at the end of the script. The ratings were given on the following scale: below expectations (1), questionable (2), meets expectations (3), exceeds expectations (4). Table A1 shows the summary of their first impression ratings and Table A2 of their final ratings. A "good" performance was considered successfully scripted if the reviewers' scores consisted of mostly "exceeds expectations" (4) and a "bad" performance if the scores were generally between "below expectations" (1) and "questionable" (2). Based on the reviewers' feedback, some minor adjustments to the scripts were made. For script A, the start was slightly worsened, whereas the rest of the student performance was made better to highlight the ascending performance. An example of a worsened performance is making the student less empathetic, e.g. not saying "I'm sorry to hear this" when confronted with severe patient complaints. No changes were deemed necessary for script B. For script C and D the start of the student performance was made better to represent a "good" start.

Table A1. Summary Review First Impression per Script.

	Ascending Performance (aim: poor first impression)		Descending Performance (aim: good first impression)	
	Script A	Script B	Script C	Script D
R1	1	2	3	2
R2	2	2	4	2
R3	2	1	3	2
R4	3	3	3	3
R5	3	2	3	4
R6	-	2	3	3

Note. Scripts: A cough, B stomach complaints, C headache complaints, D tiredness.
R=Reviewer
Ratings: 1=below expectations, 2=questionable, 3=meets expectations, 4=exceeds expectations.

Table A2. Summary Review Final Rating per Script.				
	Ascending Performance (aim: good final rating)		Descending Performance (aim: poor final rating)	
	Script A	Script B	Script C	Script D
R1	3	3	1	1
R2	3	4	1	1
R3	2	3	1	1
R4	3	3	2	2
R5	3	4	2	1
R6	3	4	2	1
Notes. Scripts: A cough, B stomach complaints, C headache complaints, D tiredness. R=Reviewer<br>Ratings: 1=below expectations, 2=questionable, 3=meets expectations, 4=exceeds expectations.				

After rating the student performances, the reviewers were asked to give written feedback to elaborate on their ratings, as well as rate the scripts on their authenticity. This rating ranged from 1 "script is very unrealistic" to 7 "script is very realistic" (see Table A3 for the summary). With very few exceptions, the authenticity ratings were relatively high. The reviewers that gave lower ratings were concerned about the performance change. As this was intended by the research team and could therefore not be adjusted, the authenticity ratings were deemed acceptable.

Table A3. Summary Review Authenticity per Script.				
	Ascending Performance		Descending Performance	
	Script A	Script B	Script C	Script D
R1	5	6	5	6
R2	6	4	3	6
R3	5	2	6	6
R4	5	4	6	5
R5	6	3	2	5
R6	6	5	4	6
Notes. Scripts: A cough, B stomach complaints, C headache complaints, D tiredness. R=Reviewer.<br>Ratings from 1 ("script is very unrealistic") to 7 ("script is very realistic").				

Appendix B

Table B1 Participant demographics

		Group 1 (N=35)	Group 2 (N=46)	Significance (p) Chi-square/ANOVA
Function: specialists / residents, N (%)		18 (51 %) / 17 (49%)	26 (57%) / 20 (43%)	0.66 (Fisher exact)
Gender: male/female, N (%)		16 (46%) / 19 (54%)	19 (42%)/ 26 (58%)[1]	0.82 (Fisher exact)
Participant ethnicity: Europe[2]/ outside Europe[3], N (%)		2 (6%)	5 (11%)	0.69 (Fisher exact)
Age in years, mean N (95% CI)		40.32 (36.0.0-44.6)[1]	37.5 (34.5-40.6)[1]	0.28
Years of experience (in their current function), mean N (95% CI)		8.3 (5.6-10.8)	6.1 (3.9-8.2)	0.22
Years of experience (evaluating trainees), mean N (95% CI)		8.2 (7.0-11.9)	7.6 (5.6-9.6)	0.71
Notes. 95% CI = 95% confidence interval.<br>[1] one individual did not disclose<br>[2] includes Netherlands, [3] one participant reported a Turkish origin				

Appendix C

Table C1. Linear Mixed Model Estimates of Hypothesis 1-4.

Variables	First Impression (H1)		Final Rating (H2)		Rating Changes (H3)		Confidence (H4)	
	Ascending Performance	Descending Performance	Ascending Performance	Descending Performance	Ascending Performance	Descending Performance	Ascending Performance	Descending Performance
Intercept	5.539** [5.241, 5.837]	7.476** [7.260, 7.692]	7.684** [7.414, 7.953]	4.313** [3.944, 4.681]	2.174** [1.880, 2.468]	3.163** [2.786, 3.541]	5.206** [4.946, 5.465]	5.387** [5.171, 5.602]
Script[1]	-1.253** [-1.527, -0.980]	-0.207 [-0.432, 0.017]	-0.573** [-0.827, -0.319]	-0.725** [-0.997, -0.452]	0.716** [0.448, 0.983]	0.517** [0.201, 0.834]	0.138 [-0.067, 0.343]	0.015 [-0.165, 0.195]
Ethnicity	0.225 [-0.048, 0.498]	-0.164 [-0.389, 0.061]	-0.312* [-0.567, -0.058]	0.210 [-0.062, 0.483]	-0.458** [-0.725, -0.191]	-0.374* [-0.691, -0.058]	-0.166 [-0.067, 0.343]	-0.072 [-0.252, 0.108]
R^2	.578	.219	.449	.492	.531	.584	0.628	0.563

Notes. ** p<.01, * p<.05.

[1] Ascending Performance: Script A vs. B. Descending Performance: Script C vs. D.

Appendix D, Table D1. Means, Standard Deviations, Bivariate Correlations, and Reliabilities (in Brackets).

			M	SD	1	2	3	4	5	6	7	8	9	10	11	12	13	14	15	16	17	18	19	20	21	22	23
	1	Age	38.75	11.14	--																						
	2	Gender	.56	.50	-.10	--																					
	3	Function	.46	.50	.77**	-.06	--																				
	4	NFC	3.35	.54	-.25*	-.00	-.22	(.77)																			
	5	SDO	1.91	.62	.02	-.14	.08	.06	(.74)																		
	6	EMS	2.37	.81	-.28*	.14	-.12	.23*	.12	(.78)																	
	7	IMS	3.95	.64	-.11	.15	-.19	.00	-.44**	-.03	(.66)																
Ascending Performance	8	FI Maj	5.22	1.27	-.26*	.12	-.18	.18	-.07	-.05	.10	--															
	9	FI Min	4.83	1.36	-.12	.08	-.09	.18	-.01	-.02	.11	.12	--														
	10	FR Maj	7.12	1.20	.11	.09	.11	-.01	.03	-.21	.06	.42**	-.07	--													
	11	FR Min	7.36	.94	-.16	.09	-.17	.30**	.05	.12	.01	.11	.33**	.32**	--												
	12	Diff Maj	2.02	1.13	.35**	-.03	.27*	-.22	.09	-.07	-.08	-.66**	-.27*	.33**	.17	--											
	13	Diff Min	2.58	1.27	-.01	-.03	.01	.05	-.02	.14	-.05	-.06	-.79**	.24*	.24*	.34**	--										
	14	Conf Maj	5.10	1.14	.24*	-.15	.16	-.03	.17	-.08	-.24*	-.21	-.14	.04	.00	.24*	.08	--									
	15	Conf Min	5.28	.98	.27*	-.14	.14	.05	.11	-.10	-.16	-.06	-.29**	.10	-.07	.16	.17	.63**	--								
Descending Performance	16	FI Maj	7.22	.81	-.10	.06	-.07	.09	.02	-.04	.10	.18	.02	.10	.17	-.13	.09	-.09	.03	--							
	17	FI Min	7.36	.80	-.34**	-.09	-.29**	.15	.09	.01	-.05	.17	.35**	.07	.26*	-.16	-.25*	-.01	-.02	.19	--						
	18	FR Maj	4.21	1.55	-.07	.04	-.13	.32**	.05	-.07	.06	.46**	.39**	.01	-.01	-.50**	-.43**	-.08	.04	.32**	.10	--					
	19	FR Min	3.90	1.59	-.15	.14	-.18	.25*	.15	-.04	.07	.26*	.62**	-.08	.09	-.36**	-.59**	.01	-.03	.17	.27*	.60**	--				
	20	Diff Maj	3.01	1.50	.02	-.01	.09	-.28*	-.04	.05	-.02	-.38**	-.39**	.05	.10	.44**	.50**	.04	-.03	.21	-.00	-.86**	-.52**	--			
	21	Diff Min	3.46	1.57	-.02	-.18	.03	-.18	-.11	.04	-.10	-.18	-.45**	.12	.04	.28*	.47**	-.02	.02	-.08	.24*	-.55**	-.87**	.52**	--		
	22	Conf Maj	5.32	.88	.19	-.20	.17	.07	.02	-.14	-.11	.07	-.09	.12	-.02	-.02	.11	.39**	.52**	.39**	-.08	.26*	.07	-.06	-.11	--	
	23	Conf Min	5.40	.85	.15	-.19	.07	.10	.03	-.04	-.19	.05	-.16	.06	-.04	-.01	.03	.53**	.54**	.14	-.05	.11	.00	-.03	-.03	.57**	--

Notes. FI = First Impression, FR = Final Rating, Maj = Ethnic Majority Student, Min = Ethnic Minority Student, Diff = Rating Change First Impression and Final Rating, Conf = Confidence in First Impression Rating.
** *p*<.01, * *p*<.05.
